# The Effects of Freshwater Clam (*Corbicula fluminea*) Extract on Activated Hepatic Stellate Cells

**DOI:** 10.1155/2021/6065168

**Published:** 2021-11-12

**Authors:** Shou-Lun Lee, Wei-Hsiang Hsu, Chia-Ming Tu, Wen-Han Wang, Cheng-Yao Yang, Hsien-Kuang Lee, Ting-Yu Chin

**Affiliations:** ^1^Department of Biological Science and Technology, China Medical University, Taichung, Taiwan; ^2^Department of Bioscience Technology, Chung Yuan Christian University, Taoyuan, Taiwan; ^3^Graduate Institute of Veterinary Pathobiology, National Chung Hsing University, Taichung, Taiwan; ^4^Department of Anesthesiology, Chang Bing Show-Chwan Memorial Hospital, Changhua, Taiwan; ^5^Department of Chemistry, Chung Yuan Christian University, Taoyuan, Taiwan; ^6^Center for Nano Technology, Chung Yuan Christian University, Taoyuan, Taiwan

## Abstract

**Background:**

The extract of freshwater clams has been used to protect the body against liver diseases in traditional folk medicine. This study aims at investigating the effects of freshwater clam extract on activated hepatic stellate cells (aHSCs), which are critical contributors to liver fibrosis.

**Methods:**

The aHSCs used in this study were derived from hepatic stellate cells that were isolated and purified from the livers of male Wistar rats and then transformed into the activated phenotype by culturing on uncoated plastic dishes. Freshwater clam extract (CE) was collected after the outflow from the live freshwater clams in a water bath at 100°C for 60 min. The effects of CE on aHSCs were analyzed by MTT assay, flow cytometry, Oil Red O (ORO) staining, western blot, and real-time RT-PCR.

**Results:**

The results indicated that CE suppressed the proliferation of aHSCs through G0/G1 cell cycle arrest by downregulating cyclin D1 and upregulating p27. The expression levels of *a*-SMA, collagen I, TGF-*β*, and TNF-*α* were inhibited in the CE-treated aHSCs. In addition, the CE treatment increased the lipid contents in aHSCs by promoting PPAR*γ* expression. Furthermore, CE modulated the expression of ECM-related genes, i.e., by upregulating MMP-9 and downregulating TIMP-II.

**Conclusions:**

These data revealed that CE could induce the deactivation of aHSCs. We therefore suggest that CE has potential as an adjuvant therapeutic agent against hepatic fibrosis.

## 1. Introduction

Liver fibrosis is the precursor of cirrhosis causing the final stage of various chronic liver diseases and is characterized by an excessive accumulation of the extracellular matrix (ECM), which is derived from various types of myofibroblasts, such as activated hepatic stellate cells (aHSCs), periportal myofibroblasts, and bone marrow-derived fibrocytes [1, 2]. However, aHSCs have been accepted as the primary cell type in the pathogenesis of liver fibrosis [3]. HSCs play important physiological functions, such as the storage of retinoids, maintenance of hepatic architecture through the remodeling of the ECM, regulation of blood flow by contractility, and production of growth factors and cytokines [4–6]. When an acute or chronic liver injury occurs, HSCs undergo an activation process in which they transform from the quiescent state (qHSCs) into the activated state (aHSCs). aHSCs are characterized by increased proliferation, loss of retinoid storage, the expression of *a*-smooth muscle actin (*α*-SMA), and the secretion of profibrogenic mediators and ECM proteins [3, 5]. In addition, aHSCs also inhibit hepatocyte regeneration [7]. Therefore, some treatment strategies for liver fibrosis induce the apoptosis of aHSCs and/or the reversion of aHSCs into qHSCs [3].

Freshwater clams (*Corbicula fluminea*) are a common edible shellfish in some East Asian countries. The effects of freshwater clams on hepatoprotection were mentioned in a traditional Chinese medical book, Ben Cao Gamg Mu. Therefore, freshwater clam extract has been used to protect against liver diseases in traditional folk medicine. Hsu et al. reported that rats were given both CCl_4_ and freshwater clam extract simultaneously for 8 weeks, and the clam extract showed a protective ability against CCl_4_ intoxication by restoring hepatic damage markers (aspartate aminotransferase and alanine aminotransferase) and reducing the formation of malondialdehyde [8]. Freshwater clam extract alleviates nonalcoholic fatty liver disease, ameliorates exhaustive exercise induced liver, and improves hemorrhage-induced acute liver injury [9–11]. In addition, a number of studies have found that freshwater clam extract possesses various biologically active properties, including anticancer [12–14], antihypertensive [15], anti-inflammatory [10, 11, 16, 17], hepatoprotective [8–11], hypocholesterolemic, and hypolipidemic effects [9, 17–21]. The composition of freshwater clam extract, which includes free amino acids, peptides, proteins, carotenoids, phytosterols, and polysaccharides, has been reported in previous studies [13, 14, 20–25]. Abergel et al. reported that palmitic acid-induced HSC deactivation resulted in suppressed cell growth and reduced the expression of both *a*-SMA and collagen I in the rat hepatic stellate cell line PAV-1 [26].

However, no investigation has demonstrated the effect of freshwater clam extract on aHSCs. This study aimed to determine the potential antihepatic efficacy of freshwater clam extract-treated aHSCs and to provide evidence that freshwater clam extract mediates cell proliferation and the expression of hepatic fibrosis markers and inflammation-associated cytokines, and cellular lipids. We demonstrated that freshwater clam extract efficiently induced the deactivation of aHSCs.

## 2. Materials and Methods

### 2.1. Chemicals and Reagents

All chemicals were reagent-grade or higher and were obtained from Sigma-Aldrich (St. Louis, Missouri, USA) except those specified. Pronase was obtained from Roche (Indianapolis, IN, USA). Rompun 2% injection was obtained from Bayer (Yongin, Kyonggi-do, Korea). Trypan blue solution (0.5%) was obtained from Biowest (Biowest, Nuaille, France). Zoletil 50 was obtained from VIRBAC (Virbac, Carros, France). Fetal bovine serum (FBS) was obtained from Thermo Fisher Scientific Inc. (Waltham, NY, USA). All other cell culture reagents were obtained from Invitrogen (Carlsbad, CA, USA).

### 2.2. Preparation of Freshwater Clam Extract

Freshwater clams (*Corbicula fluminea*, also termed Asian clam and golden clam) used in this study were derived from Hualien County, Taiwan. The live freshwater clams were placed in beakers in a water bath at 100°C for 60 min; then, the shells opened and released an outflow, which was collected as the clam extract (CE). Next, the samples were filtered through an 80-mesh filter, and the CE was lyophilized and stored at −20°C. One hundred grams of freshwater clams could release 25 ± 5 ml outflow, which was lyophilized to obtain 0.64 ± 0.03 g CE powder.

### 2.3. Animal Preparation

Male Wistar rats were purchased from BioLASCO Taiwan Co., Ltd. (Taipei, Taiwan) with body weight between 200 and 250 g. The rats were housed in an animal facility maintained with a temperature of 22°C and relative humidity of 60% and in conditions of 12 h light-dark cycle. Animals were fed standard chow and water ad libitum. Animal experimental protocol used in this was approved by the China Medical University Institutional Animal Care and Use Committee (protocol no. 98-61-N).

#### 2.3.1. Animal Model

The rodent intragastric infusion model was used in this study with modifications [27]. In brief, male Wistar rats were implanted with catheters under anesthesia achieved with Zoletil 50 (50 mg/kg) and Rompun 2% injections (0.1 ml/kg). The catheter tip was inserted in the middle of the forestomach, and the other end of the catheter was inserted laterally to the abdominal wall, leading to the dorsal midline of the neck, where a Dacron mesh button tether connected to an Instech infusion kit was implanted (Instech Laboratories, PA, USA). Following surgery, the rats were injected subcutaneously with 5 mg/kg Rimadyl (once a day) for three days. After the wounds had completely healed, 0.5 ml 20% (v/v) CCl_4_/corn oil or corn oil was infused into the stomachs of the rats twice a week for nine weeks, followed by infusions of 2 ml outflow of freshwater clam extract (0.05 g CE) or water every day for four weeks. Next, the rats were sacrificed by asphyxiation with carbon dioxide, and their livers were removed and divided; some livers were quickly frozen in liquid nitrogen and stored at −80°C, while others were fixed in a 10% neutral buffered formalin.

#### 2.3.2. Histological Analysis

The livers were fixed in 10% neutral buffered formalin, embedded in paraffin and sectioned, and then stained with hematoxylin and eosin (H&E) or Masson's trichrome stain.

### 2.4. Hepatic Stellate Cells Isolation

The primary hepatic stellate cells (HSCs) were isolated from rat liver by perfusion of the liver with digestive enzyme and density gradient centrifugation as described previously [28]. In brief, a rat liver was placed in HBSS containing 0.01% DNase I, then using forceps mildly torn liver. The cell suspension was passed through a sterile nylon mesh. The cell suspension was centrifuged at 50 × g for 7 min at 4°C, followed by resuspended in HBSS. Finally, HSCs were purified by density gradient centrifugation using Nycodenz and Percoll. Primary HSCs were grown in Dulbecco's modified Eagle's medium (DMEM, low glucose) supplemented with 10% (v/v) FBS and 1% (v/v) penicillin/streptomycin in plates (10^5^ cells/mL) at 37°C in a 5% CO_2_-humidified atmosphere. The medium was changed every two days. The cell viability was over 90% after cell isolation. The cell purity was more than 90% as assessed by vitamin A autofluorescence.

### 2.5. Cell Culture and Treatment

The detailed culture procedure was described previously by Lee et al. [28]. Culturing HSCs on uncoated plastic plates can be spontaneously activated. aHSCs were identified by immunohistochemical staining for *a*-SMA, and the presence of cells in the activated state was greater than 95%. The aHSCs were used for the experiments at passages 1–3 and cell viability was assessed by a MTT metabolic analysis. The aHSCs were treated with 0.5, 1, and 2 mg/ml CE (or 75 *μ*M palmitic acid) at the indicated times. The cells were treated with sequential treatment of CE (or PA) every 48 h for 5 days. Next, MTT was added to the cell medium and incubated for 4 h at 37°C, and the insoluble formazan salt was dissolved in isopropanol and measured at 570 nm with an ELISA reader.

### 2.6. Cell Cycle Analysis

A previously described method was used with modifications [28]. aHSCs were cultured in 6-well plates at a density of 2 × 10^5^ cells/well in the presence or absence of CE for 5 days. After trypsinization, aHSCs were fixed in ice-cold 75% ethanol overnight at −20°C. Then, the cells were washed twice and incubated with PBS containing 20 *μ*g/mL propidium iodide, 0.1% Triton-X 100, and 100 *μ*g/ml ribonuclease A for DNA staining in the dark for 30 min. Finally, cellular DNA content was measured by flow cytometry.

### 2.7. Oil Red O Staining

The method was described with modifications [28]. The aHSCs were fixed in 10% neutral buffered formalin for 15 min and then washed with H_2_O and 60% isopropanol. Next, cytoplasmic lipid droplets were stained with 0.3% (w/v) Oil Red O in isopropanol and then washed with H_2_O until the background became clear. Next, the nuclei were stained with hematoxylin. Finally, the Oil Red O in the cytoplasm was dissolved in isopropanol, and then the absorbance was measured at 492 nm using a spectrophotometer.

### 2.8. Western Blot Analysis

As described previously with modifications [28], the aHSCs were lysed in a lysis buffer (Sigma-Aldrich, Missouri, USA). The lysed cells were centrifuged at 15,000 rpm for 15 min at 4°C; then, the supernatants (30 *μ*g protein) were loaded to SDS-PAGE and transferred onto a PVDF membrane. The membrane was blocked using 1% BSA in PBS containing 0.1% Tween-20 for 1 h at room temperature. The proteins were detected through an overnight incubation at 4°C with primary antibodies against *a*-SMA (mouse monoclonal), *β*-actin (mouse monoclonal) (Sigma-Aldrich, Missouri, USA), collagen I (mouse monoclonal), cyclin D-1 (rabbit polyclonal), p27 (rabbit polyclonal), SREBP1 (mouse monoclonal) (Santa Cruz Biotechnology, CA), and PPAR*γ* (rabbit monoclonal) (Cell Signaling Technology, Beverly, MA). Finally, the membrane was incubated with a HRP-conjugated secondary antibody for 1 h. An enhanced chemiluminescence kit (Millipore, Bedford, MA) was used to visualize the reactive signals, quantified by ImageJ software.

### 2.9. Real-Time Quantitative Reverse Transcription Polymerase Chain Reaction (qRT-PCR)

As described previously with modifications [28]. In brief, the aHSCs were lysed in TRIzol Reagent, and total RNA was extracted according to the manufacturer's protocol (Invitrogen, Carlsbad, CA, USA). A SuperScript II Reverse Transcriptase Kit (Invitrogen, Carlsbad, CA, USA) was used to synthesize cDNA from a total RNA (1 *µ*g). A Maxima SYBR Green qPCR Master Mix Kit (Thermo Fisher Scientific Inc., Vilnius, Lithuania) was used for qPCR analysis. The conditions of the qPCR analysis included an initial denaturation at 94°C for 1 min, followed by exposure to 98°C for 15 s and 60°C for 40 cycles. The primers used in this study were as follows: collagen I, forward 5′-gaaacctgatgtatgcttga-3′ and reverse 5′-gaccaggaggaccaggaagt-3'; MMP-9, forward 5′-cccacatttgacgtccagagaagaa-3′ and reverse 5′-gtttttgatgctattgctgagatcca-3'; TGF-*β*1, forward 5′-tgagtggctgtcttttgacg-3′ and reverse 5′-acttccaacccaggtccttc-3'; TNF-a, forward 5′-ggcaggtctactttggagtcattg-3′ and reverse 5′-acattccgggatccagtgagttccg-3'; TIMP-II, forward 5′-tgccctgggacacgctta-3′ and reverse 5′-gtaccacgcgcaagaaccat-3'; GAPDH, forward 5′-accacagtccatgccatcac-3′ and reverse 5′-tccaccaccctgttgctgta-3'. The reactions were measured using an ABI PRISM® 7900HT Sequence Detection System (Applied Biosystems, Foster City, CA, USA).

### 2.10. Statistical Analyses

Data are presented as the mean ± standard deviation (SD) for three independent experiments isolated from different batches of aHSCs. Statistical analysis was assessed using the unpaired Student's *t*-test for comparison of independent means. Significance was defined as follows: ^∗^*P* < 0.05 and ^∗∗^*P* < 0.01 vs. the appropriate control group. Statistical analysis was performed using SigmaPlot software (version 11).

## 3. Results

### 3.1. Antiproliferative Effect of Freshwater Clam Extract on aHSCs

**Figure 1:**
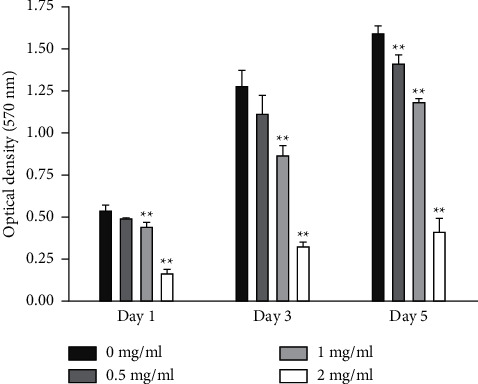
CE inhibits cell growth of aHSCs. aHSCs were incubated with clam extract (CE; 0, 0.5, 1, and 2 mg/ml) for 1, 3, and 5 days. The cell viability was determined by the MTT assay. The data are shown as the mean ± SD obtained from three independent experiments using isolated different batches of aHSCs. ^∗∗^*P* < 0.05, compared with 0 mg/ml CE.

**Figure 2:**
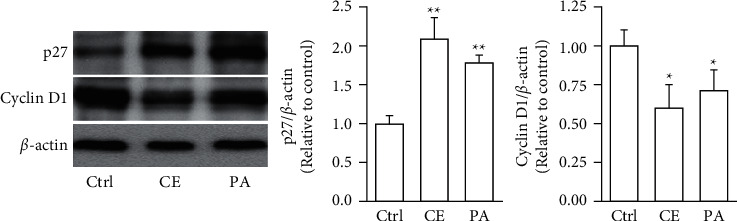
CE modulates the expression of cell cycle-related proteins in aHSCs. The aHSCs were treated with CE (1 mg/ml) and palmitic acid (PA; 75 *μ*M) for 5 days. Western blot analysis was performed to detect the level of p27, cyclin D1, and *β*-actin. A quantitative western blot analysis was used to calculate the p27 and cyclin D1 values normalized to *β*-actin. The data are shown as the mean ± SD obtained from three independent experiments using isolated different batches of aHSCs. ^∗^*P* < 0.05, ^∗∗^*P* < 0.01, compared with the control (Ctrl).

The aHSCs were treated with 0.5, 1, and 2 mg/ml freshwater clam extract (CE) for 1, 3, or 5 days. Compared to the control group, the growth of aHSCs was significantly inhibited by CE in a dose-dependent manner, as determined by the MTT assays (Figure 1). The results indicated that CE possessed an antiproliferative effect. In addition, the cell cycle analysis conducted via flow cytometry showed that the percentage of cells in the G0/G1 phase increased from 64% in the control cells to 75% in the cells treated with 1 mg/ml CE (Table 1). Moreover, a past report demonstrated that palmitic-acid- (PA-) treated PAV-1 (an HSC line) resulted in antiproliferation and induced G0/G1 arrest [26]. Herein, we investigated the effects of CE on cell cycle regulatory proteins that induced G0/G1 phase arrest. The results indicated that both CE- and PA-treated aHSCs significantly increased and reduced the expression of p27 and cyclin D1, respectively (Figure 2). Therefore, CE could induce G0/G1 cell cycle arrest by increasing p27 expression and reducing cyclin D1 expression, resulting in inhibited aHSC proliferation.

### 3.2. Effect of Freshwater Clam Extract on Deactivation of aHSCs

**Figure 3:**
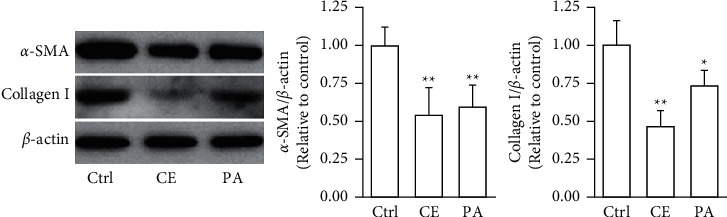
CE reduces the expression of *a*-SMA and collagen I in aHSCs. The aHSCs were exposed to CE (1 mg/ml) and palmitic acid (PA; 75 *μ*M) for 5 days. Western blot analysis was performed to detect the levels of *a*-SMA, collagen I, and *β*-actin. A quantitative western blot analysis was used to calculate the *a*-SMA and collagen I values normalized to *β*-actin. The data are shown as the mean ± SD obtained from three independent experiments using isolated different batches of aHSCs. ^∗^*P* < 0.05, ^∗∗^*P* < 0.01, compared with the control.

**Figure 4:**
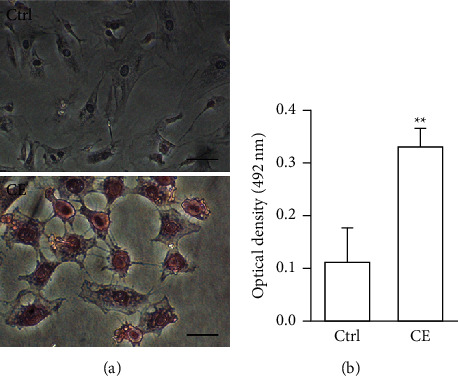
CE increases lipid content in aHSCs. The aHSCs were treated with or without CE (1 mg/ml) for 5 days and were then stained with Oil Red O and hematoxylin. Next, the cells were observed under a microscope (a). Stained Oil Red O was eluted with isopropanol and quantified by absorbance measurements at 492 nm (b). The data are shown as the mean ± SD obtained from three independent experiments using isolated different batches of aHSCs. ^∗∗^*P* < 0.01, compared with the control. The scale bar equals 20 *μ*m.

**Figure 5:**
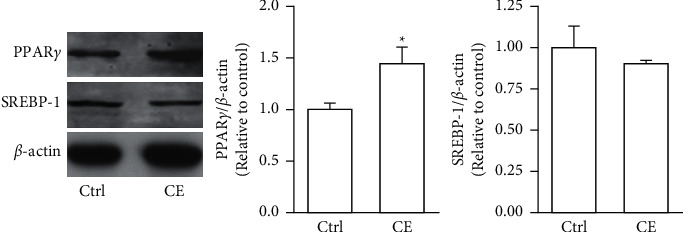
CE regulates the expression of PPAR*γ* and SREBP-1 in aHSC. The aHSCs were treated with or without CE (1 mg/ml) for 5 days. Western blot analysis was performed to detect the levels of PPAR*γ*, SREBP-1, and *β*-actin. A quantitative western blot analysis was used to calculate the PPAR*γ* and SREBP-1 values normalized to *β*-actin. The data are shown as the mean ± SD obtained from three independent experiments using isolated different batches of aHSCs. ^∗^*P* < 0.05, compared with the control.

Both *a*-SMA and collagen I are considered markers of aHSCs. As shown in Figure 3, aHSCs treated with CE and PA showed significantly reduced *a*-SMA and collagen I expression, respectively. Additionally, oil droplet depletion is another characteristic of aHSCs. In this study, aHSCs were treated with CE for 5 days and then stained with Oil Red O. The results indicated that CE significantly elevated the oil content by 3-fold compared with the oil contents of the cells without CE treatment (Figure 4). Therefore, CE had an elevated effect on the lipogenesis of aHSCs. Lipogenesis is known to be regulated by critical transcription factors, e.g., PPARs and SREBPs. We examined PPAR*γ* and SREBP-1 in aHSCs with and without CE treatment by western blot analysis (Figure 5). The results showed that the CE-induced expression of PPAR*γ* was significantly increased compared to that in aHSCs that did not undergo CE treatment. However, SREBP-1 did not differ between the treatments with and without CE. Based on the findings described above, CE can induce the deactivation of aHSCs.

### 3.3. Effects of Freshwater Clam Extract on Extracellular-Associated Gene Expression in aHSCs

**Figure 6:**
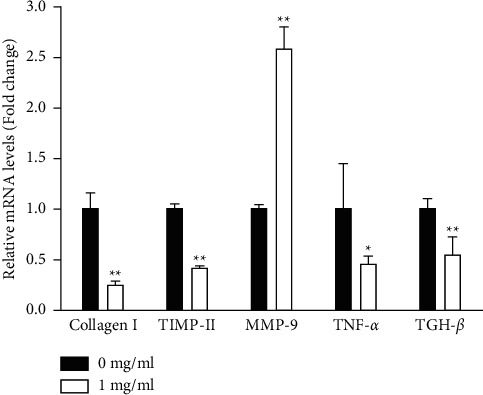
CE modulates the expression of ECM-related genes in aHSCs. The aHSCs were incubated with or without CE (1 mg/ml) for 5 days. The RNA was isolated, and the expression levels of collagen I TIMP-II, MMP-9, TNF-*α*, and TGF-*β*1 were measured by qRT-PCR. The individual mRNA levels were normalized to constitutive GAPDH expression, and the fold changes in the mRNA levels of the target genes were calculated relative to the control. The data are shown as the mean ± SD obtained from three independent experiments using isolated from different batches of aHSCs. ^∗^*P* < 0.05, ^∗∗^*P* < 0.01, compared with the control.

In this study, aHSCs were treated with CE, resulting in decreased collagen expression (Figure 3). Herein, we investigated whether CE modulated extracellular-matrix- (ECM-) associated gene expression in aHSCs. As shown in Figure 6, CE-treated aHSCs showed significantly reduced collagen I and TIMP-II mRNA levels by 76% and 58%, respectively. The CE treatment significantly increased MMP-9 in aHSCs by 2.6-fold. In addition, TNF-*α* and TGH-*β*1 have been reported to be involved in modulating the expression of ECM-related genes in aHSCs. In the CE-treated aHSCs, both TNF-*α* and TGH-*β*1, fibrogenic- and inflammation-associated genes, were significantly reduced by 55% and 45%, respectively, compared to the aHSCs that did not undergo CE treatment. Therefore, the results indicated that CE can ameliorate ECM synthesis and proinflammatory gene expression in aHSCs.

### 3.4. Regression of Liver Fibrosis by Freshwater Clam Extract in Rats with CCl_4_-Induced Liver Injury

**Figure 7:**
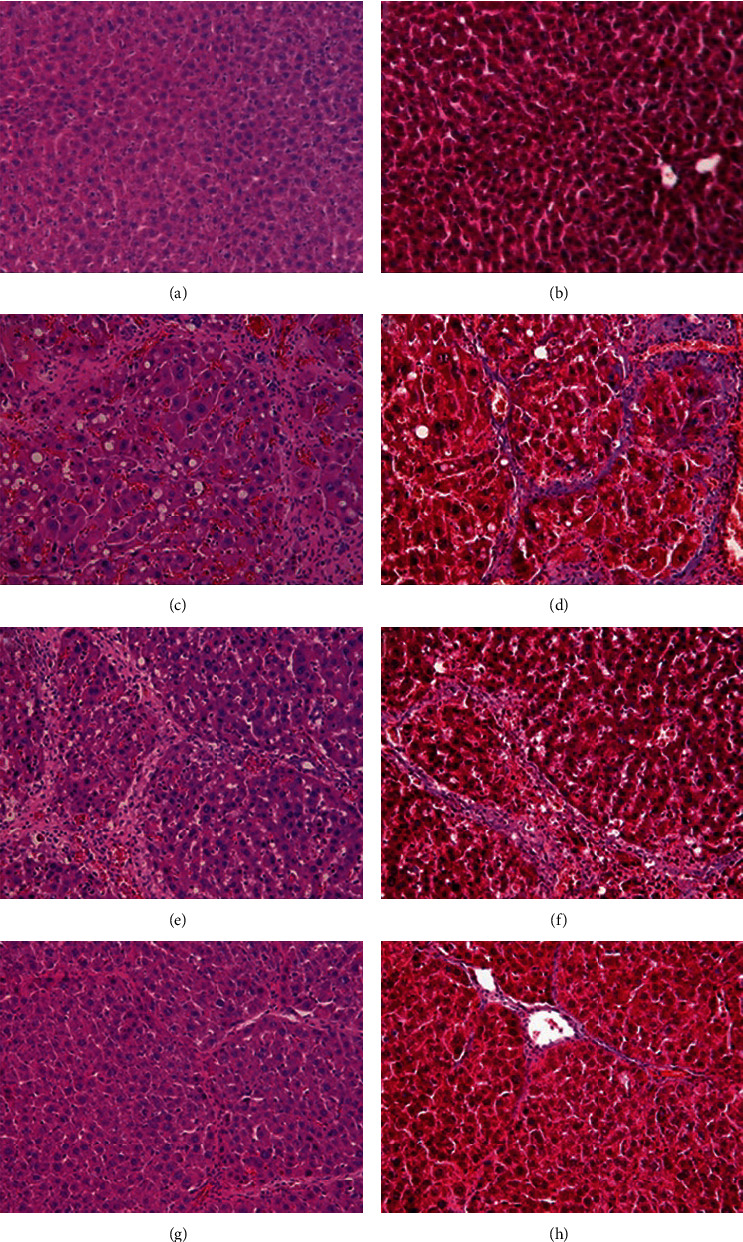
CE ameliorates hepatic fibrosis in rats with CCl_4_-induced liver damage. Wistar rats were implanted with intragastric catheters and were then infused with 0.5 ml corn oil (a, b) or 0.5 ml 20% (v/v) CCl_4_/corn oil (c, d) into their stomachs twice a week for nine weeks. Next, the CCl_4_-infused rats were infused with 2 ml water (e, f) or 2 ml CE solution (containing 0.05 g CE) (g, h) every day for four weeks. Finally, the livers were removed for a histopathological analysis after the rats were sacrificed. The liver sections were stained with H&E (a, c, e) or Masson's trichrome stain (b, d, f). Original magnification = 100 ×.

**Figure 8:**
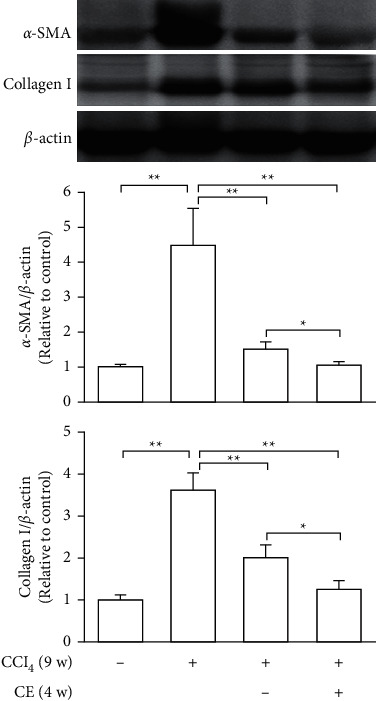
CE reduces the expression of hepatic *a*-SMA and collagen I in rats with CCl_4_-induced liver damage. Wistar rats were intragastrically infused with 0.5 ml corn oil or 0.5 ml 20% (v/v) CCl_4_/corn oil twice a week for nine weeks; then, the CCl_4_-infused rats were infused with 2 ml water or 2 ml CE solution (containing 0.05 g CE) every day for four weeks. Next, the livers were excised after the rats were sacrificed. The liver tissues were homogenized and analyzed via western blotting using anti-*α*-SMA, collagen I, and *β*-actin antibodies. A quantitative western blot analysis was used to calculate the *a*-SMA and collagen I values normalized against *β*-actin. The data are shown as the mean ± SD obtained from three independent experiments. ^∗^*P* < 0.05.

A CCl_4_-induced hepatic injury is an experimental animal model that is commonly used for liver diseases. In this study, the liver injury was analyzed using liver histology stained with hematoxylin and eosin (H&E) and Masson's trichrome (Figure 7). The rats that showed normal hepatic architecture were not treated with CCl_4_ (Figures 7(a) and 7(b)). After 9 weeks of treatment with CCl_4_, the liver tissues revealed hydropic degeneration, the infiltration of inflammatory cells, and increased septa composed of fibrosis and collagen (Figures 7(c) and 7(d). At the end of a 4-week period after the above 9-week CCl_4_ infusion, the liver tissues also revealed hydropic degeneration, inflammatory cell infiltration, and fibrous septa (Figures 7(e) and 7(f). At the end of the 4-week CE treatment, which immediately followed the 9-week CCl_4_ infusion, the liver tissues did not show hydropic degeneration, and the inflammatory cell infiltration and fibrous septa in the histology were obviously ameliorated (Figures 7(g) and 7(h). The levels of the fibrosis biomarkers *a*-SMA and collagen in the livers were detected by western blot analysis (Figure 8). The rats were infused with CCl_4_ for 9 weeks, and the *a*-SMA and collagen contents in the livers were significantly increased compared to those without CCl_4_ treatment. At the end of the 4-week treatment with or without (as the control) CE after CCl_4_ infusion for 9 weeks, the *a*-SMA and collagen I expression was significantly reduced compared to those without CE treatments in rats with CCl_4_-induced liver damage. Therefore, the results indicated that CE significantly ameliorates liver fibrosis in rats with CCl_4_-induced liver injuries.

## 4. Discussion

The hepatoprotective effect of freshwater clams has been mentioned in ancient books. Previous studies have demonstrated that freshwater clams possess many medical effects [10]. However, little information is known about the antifibrotic effect of CE on aHSCs. Our data are the first to indicate that CE treatment on aHSCs results in suppressed cell proliferation, increased lipid content, reduced expression of fibrogenic and proinflammatory cytokines, and modulated ECM formation.

### 4.1. CE Suppression of Proliferation in aHSCs

aHSC proliferation contributes to the progression of liver fibrosis. The present study showed that CE induced the inhibition of aHSC growth by the MTT assay (Figure 1). In addition, the trypan blue exclusion test and lactate dehydrogenase (LDH) cytotoxicity assay indicated that the viability of the cells was not suppressed at CE concentrations up to 1 mg/ml (data not shown). Therefore, we speculate that treating aHSCs with CE induced the inhibition of proliferation but did not induce cell death. Moreover, the CE-treated cells resulted in cell cycle arrest at the G1 phase, as determined by flow cytometric analysis (Table 1). Further analysis indicated that the CE treatment increased p27 expression and reduced cyclin D1 expression in aHSCs (Figure 2). p27 is a cell cycle inhibitor that inhibits G1 cyclin-CDK protein kinase activity [29]. Cyclin D1 is a regulator of cell cycle progression and activates CDKs in the G1 phase [30]. Thus, we speculate that CE induced the suppression of proliferation resulting from cell cycle arrest at the G1 phase through increasing p27 expression and reducing cyclin D1 expression in aHSCs. Furthermore, a report indicated that TGF-*β* induces HSC activation and proliferation [31]. Herein, our data also indicated that CE treatment reduced the TGF-*β*1 expression in aHSCs (Figure 6). These results suggest that CE could ameliorate the proliferation of aHSCs.

### 4.2. CE-Induced Deactivation of aHSCs

In addition to cell proliferation, the overexpression of *a*-SMA and collagen I, depletion of lipid droplets, and ECM remodeling are characteristics of aHSCs. The CE treatment significantly reduced the expression of *a*-SMA and collagen I in aHSCs (Figure 3). Moreover, the CE treatment increased the lipid contents in aHSCs (Figure 4). Our data also indicated that CE-treated aHSCs showed increased PPAR*γ* expression (Figure 5). However, PPAR*γ* is a transcription factor that plays a critical role in fat formation [32]. A previous study showed that PPAR*γ* expression is lower in aHSCs, but increased PPAR*γ* expression restores lipid storage in aHSCs [33]. In addition, PPAR*γ* overexpression in aHSCs results in reduced *a*-SMA and collagen synthesis and suppresses cell proliferation [32]. The activation of HSCs is an important event in the progression of liver fibrosis. Herein, our results suggest that CE could induce the deactivation of aHSCs. Seki and Brenner demonstrated that aHSCs can revert to quiescent-like HSCs when the causative factors are removed [34]. However, the mechanism by which CE-induced aHSCs convert from an activated state to a quiescent-like state will be investigated further in future work.

### 4.3. CE-Modulated ECM-Related Gene Expression

Collagen I is a main component of the ECM, and its increase is a key event in liver fibrosis. TGF-*β* is considered a marker of aHSCs and induces collagen I expression in aHSCs [35]. The results revealed that CE treatment reduced the expression of collagen I and TGF-*β* in aHSCs (Figure 6). TGF-*β*1 and TNF-*α*, fibrogenic and proinflammatory cytokines, are released from HSCs and stimulate the activation of HSCs [36]. Both cytokines modulate ECM remodeling by altering the MMP/TIMP expression [37, 38]. Our data showed that CE-treated aHSCs showed reduced cytokine expression of TGF-*β*1 and TNF-*α*. Moreover, the CE treatment induced the upregulation of MMP-9 and downregulation of TIMP II in aHSCs (Figure 6). Therefore, the results obtained herein suggest that CE can modulate ECM remodeling.

### 4.4. CE-Ameliorated CCl_4_-Induced Hepatic Fibrosis in Rats

CCl_4_-induced liver fibrosis in rodents is a widely used animal model. Rats with liver fibrosis can spontaneously recover after the cessation of the injury [39], suggesting that hepatic fibrosis is a dynamic bidirectional event. Our investigations indicated that rats previously given CCl_4_ for 9 weeks exhibited liver fibrosis; then, we continued treatment with or without CE for 4 weeks. Compared with spontaneous recovery, the CE treatments significantly reduced the expression of fibrotic markers such as *a*-SMA and collagen I (Figure 8) and improved hepatic healing (Figure 7). In addition, injection of human liver stem cells reduced the expression of *a*-SMA and collagen I resulting in attenuated nonalcoholic steatohepatitis-associated fibrosis in mice fed on a methionine-choline-deficient diet [40]. Therefore, in vitro and in vivo studies suggest that the expression of ECM-related genes can be modulated by CE.

Herein, the efficacy of CE is shown not only for inducing the deactivation of aHSCs but also for accelerating liver recovery after CCl_4_-induced liver fibrosis in rats. Thus, CE could be a potential adjuvant therapeutic agent against hepatic fibrosis. However, the deactivation of aHSCs could be caused by many factors in CE-treated aHSCs. Thus, it will be examined in the future.

## 5. Conclusions

In traditional Chinese medicine, clam extracts are thought to have hepatoprotective effects. Our results indicated that CE treatment suppressed cell proliferation, increased oil accumulation, reduced the expression of fibrogenic and proinflammatory cytokines, and changed ECM-related gene expression in aHSCs. Therefore, our results offer the first indication that CE could possess an antihepatic fibrosis effect seemingly through induced deactivation of aHSCs. These results suggest that CE could be provided as a dietary supplement for patients with hepatic fibrosis.

## Figures and Tables

**Table 1:** The cell cycle distribution of aHSCs with CE treatment.

CE (mg/ml)	SubG1	G0/*G*1	S	G2/M
0	5.7 ± 3.1%	64 ± 3%	7.7 ± 4.2%	21 ± 6%
1	4.3 ± 3.7%	75 ± 7%^∗^	5.6 ± 2.6%	15 ± 3%

Flow cytometry was used for analyzing cell cycle distribution of aHSCs with incubated presence or absence of CE for 5 days. Data are shown as the mean ± SD. ^∗^*P* < 0.05, compared with 0 mg/ml CE.

## Data Availability

The datasets supporting the conclusions of this article are included within the article.

## Abbreviations

aHSCs: Activated hepatic stellate cells
*α*-SMA: *α*-Smooth muscle actin
CE: Freshwater clam extract
ECM: Extracellular matrix
HSCs: Hepatic stellate cells
MMP: Matrix metalloproteinase
PPAR*γ*: Peroxisome proliferator-activated receptor gamma
SREBP: Sterol regulatory element-binding proteins
TGF-*β*: Transforming growth factor beta
TIMP-II: Tissue inhibitor of metalloproteinases 2
TNF-*α*: Tumor necrosis factor-alpha.
